# Toward the use of buprenorphine in infants for neonatal opioid withdrawal syndrome: summary of an NIH workshop

**DOI:** 10.1038/s41372-020-00886-7

**Published:** 2021-01-25

**Authors:** Alan E. Simon, Michelle P. Freund, Stephanie Wilson Archer, Andrew A. Bremer

**Affiliations:** 1 Office of the Director, National Institutes of Health, Bethesda, MD 20892 USA; 2 National Institute on Drug Abuse, National Institutes of Health, Bethesda, MD 20892 USA; 3 Eunice Kennedy Shriver National Institute of Child Health and Human Development, National Institutes of Health, Bethesda, MD 20892 USA

**Keywords:** Outcomes research, Public health

Opioid use by pregnant women represents a major public health concern given the impact of opioid exposure on maternal and neonatal outcomes, including increased risk of maternal mortality, preterm labor, stillbirth, and neonatal opioid withdrawal syndrome (NOWS) [1]. Unfortunately, national rates of opioid use disorder (OUD) in pregnant women at delivery hospitalization more than quadrupled during 1999–2014, and almost 7% of pregnant women used a prescription opioid in 2019 [2]. Consequently, these trends have led to a fivefold increase in cases of NOWS since 2004 [3], and this rise may be exacerbated by the COVID-19 pandemic. Recent data from the U.S. Pediatric Health Information System suggest an incidence of NOWS as high as 20 cases per 1000 live births with treatment costs of approximately $560 million per year [4, 5].

Currently, the most common first-line medication used to treat NOWS is morphine. A minority of infants are treated with methadone, and a very small percentage receive buprenorphine [6]. Despite its rare use in this capacity, a recent meta-analysis of smaller studies suggested that buprenorphine may be the optimal pharmacologic treatment for infants with NOWS [7]. However, the primary findings of this meta-analysis should be interpreted with caution; large, properly conducted, direct comparative-effectiveness studies are needed to inform clinical practice. A greater understanding of the benefits, risk, and barriers to the use of buprenorphine is needed if clinicians are to make informed decisions about its use for the treatment of NOWS.

To move the field forward, the National Institutes of Health (NIH) hosted a two-day virtual workshop in August 2020 entitled “*Toward the Use of Buprenorphine in Infants: Scientific and Practical Considerations* [8].” The workshop was supported by the NIH Helping to End Addiction Long-term^SM^ (HEAL) Initiative, a trans-agency effort to speed scientific solutions to stem the national opioid public health crisis [9]. Table 1 highlights the workshop findings; a brief description of each of the workshop’s sessions is noted below and a more comprehensive review is forthcoming.

**Table 1:** Issues to address for a randomized clinical trial recommended by workshop participants.

Study question •A randomized clinical trial is needed to compare buprenorphine, morphine, and methadone •Include all infants requiring pharmacologic care for NOWS vs. trials of subgroups of infants with maternal opioid exposure based on maternal and infant characteristics. Subgroups may be defined by maternal polysubstance use, maternal medication used for medication-assisted treatment, presence of comprehensive treatment, and infant genetics
Study design •May need a pragmatic trial design •Masking intervention arms is essential to conduct a rigorous trial, but is challenging in this context given that the study drugs have different routes of administration, dose intervals, and weaning protocols
Site- and person-level recruitment challenges •Site investigator equipoise. Sites may not be willing to conduct certain trials due to strongly held opinions about current treatment approaches and the alcohol content of the current buprenorphine preparation •Sites need to have the eligible population and a research pharmacy able to provide access to controlled-substance study drugs 24/7 •Recruiting participants may be challenging as potential participants may have concerns over privacy, mandatory reporting laws, and potential loss of child custody
Outcomes •Need to select salient short-term outcomes that are feasible to collect, which might include sleep, respiratory outcomes, gastrointestinal outcomes, and emotional dysregulation during the first year of life •Neurodevelopmental outcomes at 2 years or more of age are critical but follow-up of participants may be difficult

The first session focused on the state of the science, informed by the Advancing Clinical Trials in Neonatal Opioid Withdrawal Syndrome (ACT NOW) Current Experience study. This study of 1808 infants with NOWS, conducted at 30 U.S. sites, found considerable variability in what drugs infants were exposed to—illicit and prescription opioids, maternal medication for addiction treatment, and polysubstance exposures (e.g., tobacco, methamphetamine)—and how mothers and infants are clinically managed [6]. Furthermore, the limitations of previous NOWS-related studies were reviewed, including but not limited to issues regarding investigator blinding, variable use of non-pharmacological interventions during trials, and variation in weaning protocols tested, as well as statistical issues regarding power, pairwise comparisons, and adjustments for adjuvant therapies.

The second session focused on ongoing and upcoming clinical trials. Specifically, the ACT NOW Program, a HEAL-supported collaboration between the *Eunice Kennedy Shriver* National Institute of Child Health and Human Development and the NIH Environmental influences in Child Health Outcomes Program, is currently conducting three large studies evaluating: (i) how rapidly it is safe to wean infants with NOWS off of morphine or methadone; (ii) whether the Eat, Sleep, Console approach can shorten hospital stay more than using the Finnegan Neonatal Abstinence Scoring Tool (FNAST or modified FNAST); and (iii) what changes happen in brain development and associated neurodevelopment and behavioral outcomes in infants with NOWS. All three studies will be assessing neurodevelopment at 24 months of age. Moreover, the National Institute on Drug Abuse has embarked on the HEAL-supported HEALthy Brain and Child Development Study (HBCD). Although still in the planning phase, the HBCD Study will establish a large cohort of pregnant women from regions of the country significantly affected by the opioid crisis and follow them and their children for at least 10 years.

The third session focused on feasibility and operational issues for a potential trial including buprenorphine for the treatment of NOWS. Numerous challenges exist. Identifying the most salient research question is challenging, given the variation in experiences for mothers with OUD and infants with NOWS, including what medication-assisted treatment (MAT) the mother received, when and how long she was treated during pregnancy, whether she received comprehensive treatment versus medication only, and if she used multiple substances during her pregnancy. Testable interventions might compare the effectiveness of the primary medications used among all infants with NOWS or compare the effectiveness of matching the infant’s medication with the mother’s MAT medication. Finding sufficient study sites for future trials may also be difficult, as many potential sites may not have equipoise for all study questions. Furthermore, many sites do not have the capacity to conduct these studies, as they do not have research pharmacy access to controlled-substance study drugs 24 hours a day, 7 days a week.

The last session focused on next steps. Because current morphine, methadone, and buprenorphine formulations have much different routes of administration (oral versus sublingual), dose intervals, and weaning protocols, reliably masking study drugs is difficult. Selecting clinically meaningful neurodevelopmental and non-neurodevelopmental outcomes that allow feasible trial length and cost poses a formidable challenge as well. Because this study population may be in unstable living situations and infants are often placed in foster care, collecting long-term outcomes requires research staff to develop relationships with families, guardians, and stakeholders (e.g., foster care organizations and legal systems) to maintain contact and establish trust. Finally, several statistical considerations include selecting pragmatic, validated assessment instruments sensitive enough to quantify treatment effects and taking into account the myriad confounding factors (neonatal intensive care unit vs. special-care nurseries, varying non-pharmacologic approaches, polysubstance exposures in utero combined with post-natal adjuvant NOWS treatments, and clinical management practices).

In conclusion, considering the gaps in knowledge in whether and when to use buprenorphine to treat NOWS and the potential for better outcomes, a comparative-effectiveness, randomized controlled trial was recommended by the experts at the workshop to inform clinical practice. Numerous issues were identified during the workshop that need to be considered to design and implement this much needed study successfully.

## Disclaimer

The contents of this article represent the authors’ views and do not constitute an official position of the National Institutes of Health or the U.S. Government.
